# Combating the effects of climatic change on forests by mitigation strategies

**DOI:** 10.1186/1750-0680-5-8

**Published:** 2010-11-30

**Authors:** Michael Köhl, Rüdiger Hildebrandt, Konstantin Olschofksy, Raul Köhler, Thomas Rötzer, Tobias Mette, Hans Pretzsch, Margret Köthke, Matthias Dieter, Mengistu Abiy, Franz Makeschin, Bernhard Kenter

**Affiliations:** 1 Institute for World Forestry and Climate Campus, University of Hamburg, Leuschnerstraße 91, D-21031 Hamburg, Germany; 2 Chair of Forest Yield Science, Technical University of Munich, Hans-Carl-von-Carlowitz-Platz 2, D-85354 Freising, Germany; 3 Institute of Soil Science and Site Ecology, Faculty FGH, Dresden University of Technology, Pienner Straße 19, D-01735 Tharandt, Germany; 4 Institute of Forest Based Sector Economics, Johann Heinrich von Thünen Institute Federal Research Institute for Rural Areas, Forestry and Fisheries, Leuschnerstraße 91, D-21031 Hamburg, Germany

## Abstract

**Background:**

Forests occur across diverse biomes, each of which shows a specific composition of plant communities associated with the particular climate regimes. Predicted future climate change will have impacts on the vulnerability and productivity of forests; in some regions higher temperatures will extend the growing season and thus improve forest productivity, while changed annual precipitation patterns may show disadvantageous effects in areas, where water availability is restricted. While adaptation of forests to predicted future climate scenarios has been intensively studied, less attention was paid to mitigation strategies such as the introduction of tree species well adapted to changing environmental conditions.

**Results:**

We simulated the development of managed forest ecosystems in Germany for the time period between 2000 and 2100 under different forest management regimes and climate change scenarios. The management regimes reflect different rotation periods, harvesting intensities and species selection for reforestations. The climate change scenarios were taken from the IPCC's Special Report on Emission Scenarios (SRES). We used the scenarios A1B (rapid and successful economic development) and B1 (high level of environmental and social consciousness combined with a globally coherent approach to a more sustainable development). Our results indicate that the effects of different climate change scenarios on the future productivity and species composition of German forests are minor compared to the effects of forest management.

**Conclusions:**

The inherent natural adaptive capacity of forest ecosystems to changing environmental conditions is limited by the long life time of trees. Planting of adapted species and forest management will reduce the impact of predicted future climate change on forests.

## Background

31.5 percent of the land area in Europe (excluding the Russian Federation) is covered by forests, which provide multiple ecosystem services and functions. The carbon stored in their biomass amounts to an estimated 53 billion t C [1]. They are the single largest natural ecosystem supporting biodiversity in Europe [2] and a source of renewable energy and materials. Forest activities, wood industries and the pulp and paper industries combined contribute 1 percent of the Gross Domestic Product (GDP) in European countries [1].

Temperature and the availability of soil moisture are governing the natural range of European tree species. The structure and composition of many forests are further influenced by natural disturbance regimes, such as storm, fire, or insects. Forests have adapted to changing climatic conditions in past millennia. However, the recent, human-induced changes of climatic conditions are occurring at rates that might overcharge the natural adaptation potential of tree species [3]. Climate change scenarios indicate that in Central Europe temperatures will increase by about 3°C by 2100 [4]. The heat wave that struck European forests in 2003 might suggest an apprehension of climatic change impacts we need to expect in the future. According to ICP-Forests [5] the severe reduction of water availability led to reductions of tree vitality and tree growth and fostered bark beetle attacks in larger areas. Where forests are at the edge of their bio-geographical distribution, temperature rise may lead to increased tree mortality, especially in southern and central Europe [6-8]

On the other hand changes in climate patters might promote forest growth. Menzel and Fabian reported that the average annual growing season has lengthened by 10.8 days since the early 1960 s [9]. Under a warmer climate an increase of forests growth and yield is expected in commercial forests in northern Europe [10].

Many studies have investigated the potential impacts of climate change on the abundance and vitality of tree species. For example, in the boreal forests of northern Sweden an upward shift of the timberline has been observed for mountain birch (Betula pubescens ssp. tortuosa) and was attributed to climate change [11]. Due to milder winters in Austrian Alpine valleys seed germination and seedling survival of Walnut (Juglans regia) have been favored [12]. Hemery et al. [13] report specific risks and opportunities for scattered broadleaved species in Europe under climate change. Much less attention has so far been paid to the adaptive capacity of forests to climate change. The inherent adaptive capacity describes evolutionary mechanisms and processes that permit tree species to adjust to new environmental conditions [14]. The majority of European forests is managed [1] and thus offer the possibility to improve their adaptation ability by human intervention. Among those interventions are the selection of tree species or provenances, the regulation of rotation periods, or the implementation of management regimes that reduce the impact of biotic and abiotic hazards.

Forest activities play a key role in the context of global change [15,16]. While deforestation and degradation which take place mainly in tropical regions account for roughly 20 percent of the global carbon emissions [17], sustainable forest management activities contribute to climate change mitigation [16,18]. It is widely accepted that the carbon stock of natural forest ecosystems should be protected [19,20] and management activities be focused on reforestation, the increase of carbon stock density in existing forests, fostering the C storage in harvested wood products and substitution of fossil fuels through material and energetic use of timber [16,18,21,22].

In the following we present the results of a simulation study that demonstrate the development of German forests until 2100 under different climate change and management scenarios. Currently 99.2 percent of German forests are available for wood supply [23]. Roughly 20 percent of the forest area is managed as continuous cover forests [24], while 80 percent are even-aged forests [25]. Forest management can be focused on different objectives including profit maximization, biodiversity conservation, recreation, or protection from natural hazards. For the simulation study we did not presume current management presettings as stipulated by the national forestry programme for Germany [26], but selected three sustainable management schemes [27-30] that pursue different objectives:

### Maximum profit

The management objective is profit maximization. The final cut can be realized at minimum age of 50 years (coniferous trees) or 70 years (broadleaved trees). Forest stands are harvested when the rate of return becomes smaller than 2 percent. Tree species that guarantee maximum profit are selected for reforestation. The management scheme promotes single species, even-aged stands, harvesting by clear-cuts and artificial regeneration by planting.

### Maximum net annual forest rent

The decision about final cuts is based on the increase of the annual sustained forest rent. As long as the net annual rent is increasing forest stands are thinned but not finally cut. Reforestations are realized with species that guarantee the highest revenue at a marginal interest rate of 0 percent under the specific, local site conditions. Artificial or natural regeneration can be applied.

### Diameter limit cut

The decision for final cuts is oriented towards biological criteria and reflects close-to-nature forest management. Trees are cut when they reach either a minimum diameter or a given age. Natural regeneration with tree species of the site specific potential natural forest vegetation is assumed. This management alternative is oriented towards close-to-nature forestry, which will result in continuous cover forests with mixed-species, uneven-aged stands. In the following age-classes managed under this alternative are not presented as the mean age of all trees in a stand, but reflect the age of the oldest trees in a stand.

As future climate change cannot be predicted, climate change scenarios were selected from the IPCC Special Report on Emissions Scenarios (SRES) [31]. We applied the following SRES scenarios:

### A1B

The A1 storyline and scenario family describes a future world of very rapid economic growth, global population that peaks in mid-century and declines thereafter, and the rapid introduction of new and more efficient technologies. Major underlying themes are convergence among regions, capacity building, and increased cultural and social interactions, with a substantial reduction in regional differences in per capita income. The A1 scenario family develops into three groups which are distinguished by their technological emphasis [31]. A1B assumes a balance across fossil intensive and non-fossil energy sources. Scenario A1B will result in a steady increase of global temperatures.

### B1

The B1 storyline and scenario family describes a convergent world with the same global population that peaks in mid-century and declines thereafter, as in the A1 storyline, but with rapid changes in economic structures toward a service and information economy, with reductions in material intensity, and the introduction of clean and resource-efficient technologies. The emphasis is on global solutions to economic, social, and environmental sustainability, including improved equity, but without additional climate initiatives [31]. The scenario is oriented towards the political goal to stabilize the increase of temperatures by 2°C compared to the average pre-industrial surface temperatures [32].

## Results

The objective of our simulation was to describe the potential future development of forests in Germany under different climate change and management scenarios until 2100. The initial situation for the year 2000 was obtained from data assessed by the German national forest inventory (NFI). The German NFI is a sample-based survey that provides representative data for the entity of German forests. We utilized spatially explicit attributes such as tree species, age classes, tree dimensions, timber volumes, carbon stock, regeneration, or site productivity. Data from the year 2000 were utilized as the initial state of the simulations. The development of forests is influenced by a set of site factors such as precipitation, temperature, soil substrate, or topography. The characterization and spatial pattern of site factors is subject to local differences. In Germany a hierarchical system has been developed that summarizes the regional differences of site factors in growing regions [15]. Growing regions form regional units with a uniform physiographical character and climate. As future climate change will alter regional climates of growing regions we chose growing regions as units for spatially explicit considerations.

**Figure 1:**
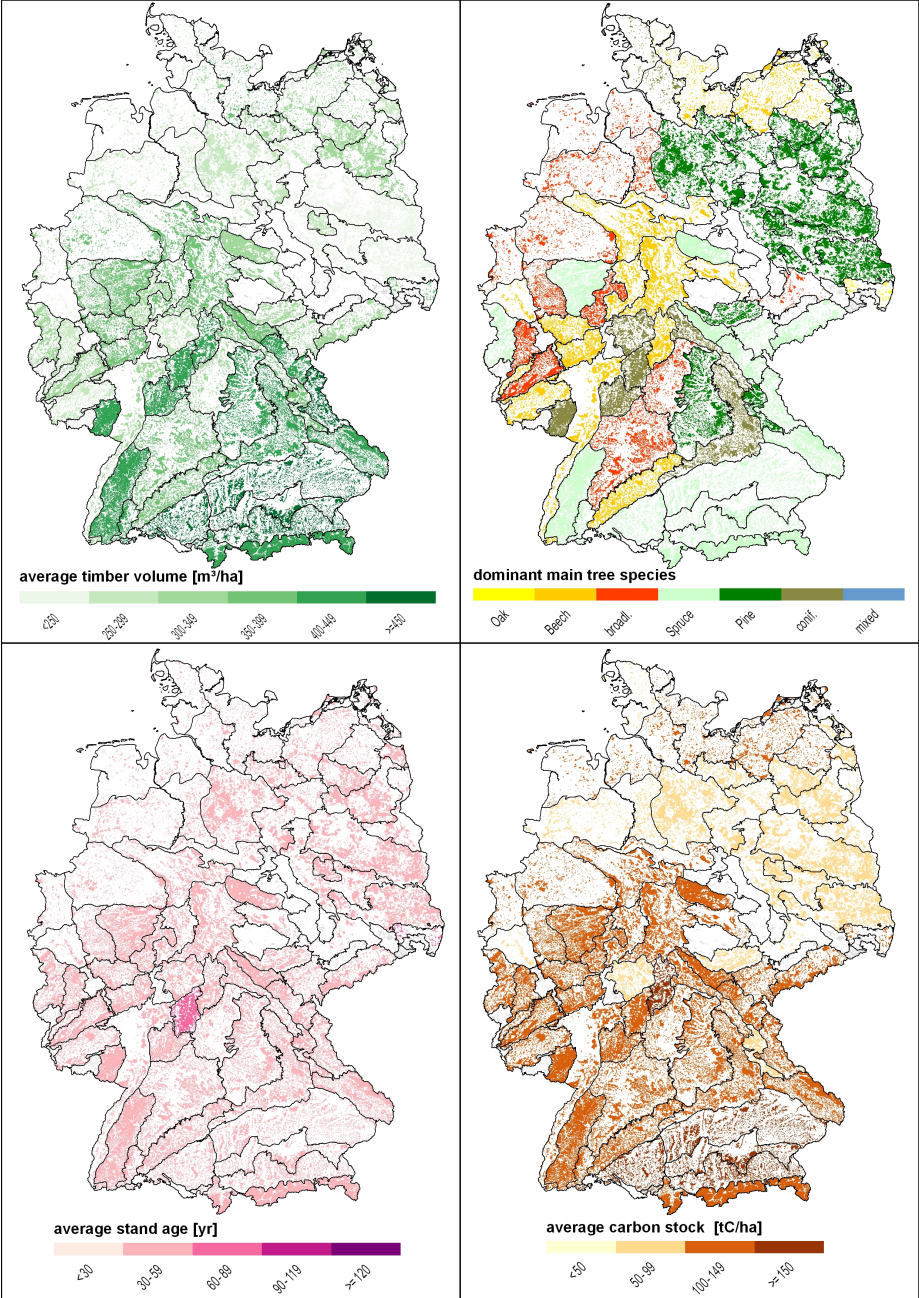
**Initial stage in 2000 for standing timber volume**. main tree species, stand age, and carbon stock by growth regions.

Figure 1 presents the initial situation in the year 2000 for the spatial distribution of standing timber volume [m^3^/ha], main tree species [spruce, pine, oak, beech, mixed species stands], stand age [in 30-year classes], and carbon stock [t/ha]. Timber volume and carbon stock are highly correlated and show in southern Germany higher values than in northern Germany. These differences are also reflected by the patterns of stand age.

**Figure 2:**
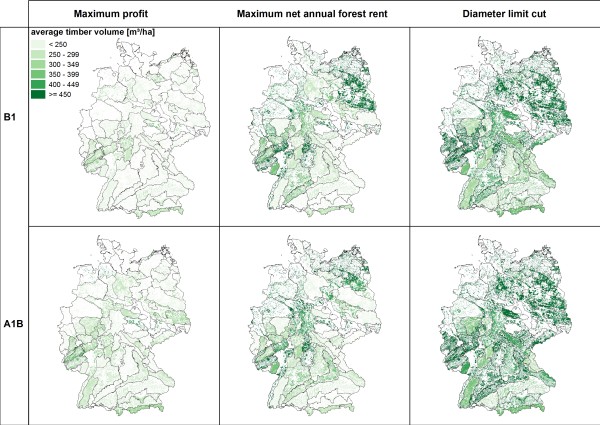
**Average standing timber volume [m3/ha] in 2100**.

**Figure 3:**
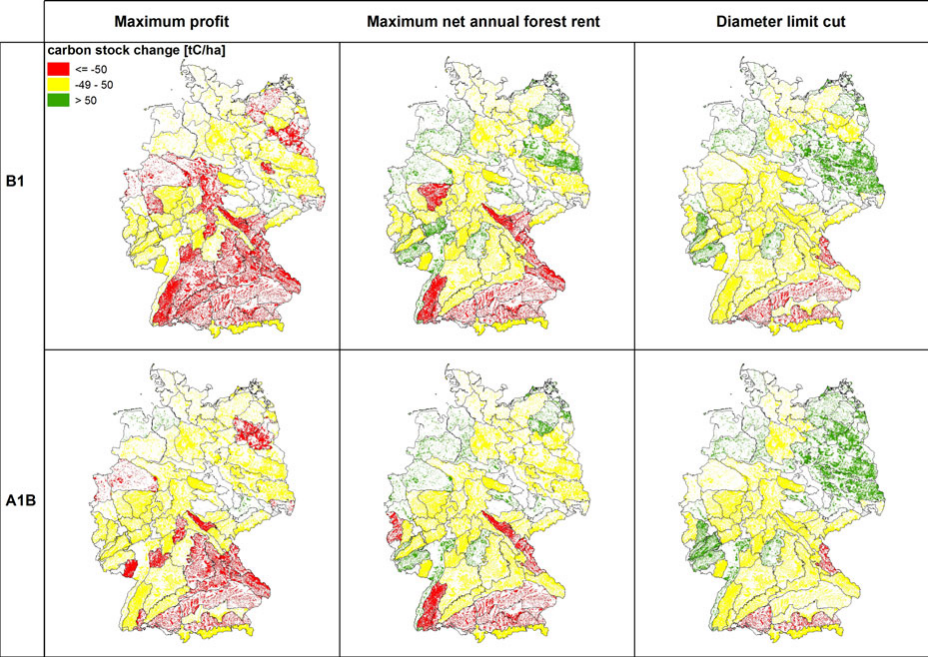
**Carbon stock change between 2000 and 2100 [< -50 tC/ha; -50 to 49 tC/ha; > 50 tC/ha]**.

**Figure 4:**
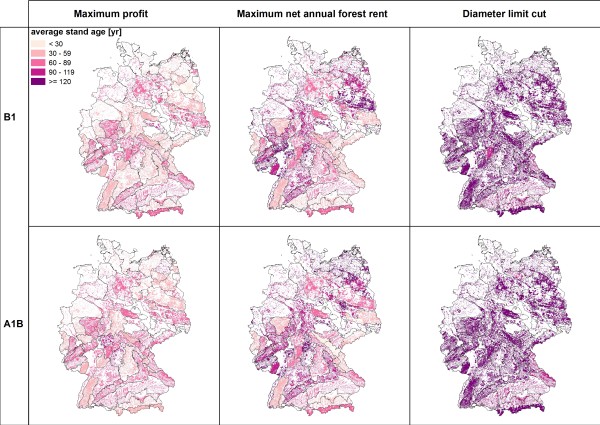
**Mean stand age 2100**.

**Figure 5:**
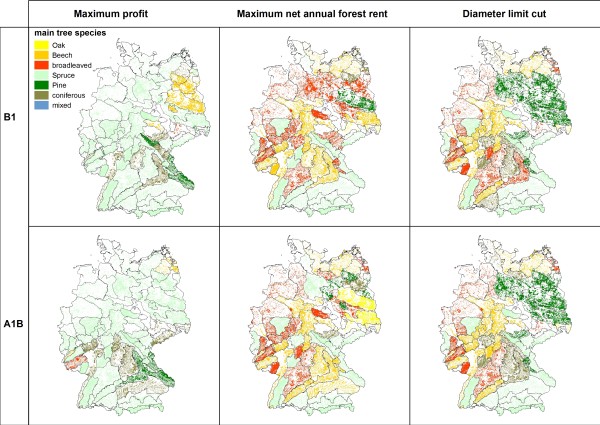
**Dominating tree species in 2100**.

Based on the initial values of the year 2000 the development of standing timber volume, carbon stock, stand age, and dominant tree species was simulated under the three management schemes and two climate change scenarios given above. In figures 2, 3, 4 and 5 the simulation results for the year 2100 are shown. All management regimes follow the principle of sustainability and do not lead to an overexploitation of forests. In 2000 the average standing timber volume of Germany equalled 320 m^3 ^per hectare, with highest values in southern Germany (Federal State of Bavaria: 402 m^3^/ha; Baden-Württemberg: 365 m^3^/ha) and lowest values in eastern Germany (Brandenburg: 239 m^3^/ha; Saxony-Anhalt: 237 m^3^/ha).

Among the three management types the simulation results for 2100 show the lowest standing timber volumes for "*maximum profit oriented management" *(figure 2). The average timber volumes are consistently below 350 m^3^/ha and indicate a substantial reduction of the initially high timber volumes in southern Germany. The extraction of timber is reflected by the changes of carbon stocks (figure 3). Under maximum profit oriented management carbon stocks remain constant or decrease. The mean age of forests stands is uniquely lower than under the other management objectives as a consequence of the high amount of final harvests and subsequent reforestations (figure 4). Reforestation is mainly realized with coniferous species and thus results in the dominance of mainly spruce and pine (figure 5). Only in north eastern Germany beech is found to be the dominating species and - under scenario A1B - some scattered regions are dominated by broadleaved species in central Germany.

Management under the objective "*diameter limit cut*" harvests trees when they reached a minimum threshold tree diameter of 45 to 80 cm in 1.3 meter height above ground. This management alternative leads to an (over)maturation of the stands and thus higher standing timber volumes (figure 2) and average stand ages (figure 4). This effect can mainly be observed in central and north eastern Germany, where the initial standing timber volumes were lower than in southern Germany. The vast majority of regions show standing timber volumes above 350 m^3^/ha. Carbon stocks remain constant or increase (figure 3), especially in north eastern and south western Germany, while decreases of carbon stock can be found in some regions in southern Germany. While in eastern and southern Germany coniferous species are dominant, broadleaved species are dominating in central and northern Germany.

Management according to the "*maximum net annual forest rent*" takes an intermediate position between the other two management alternatives. In some regions in south western and north eastern Germany average standing volumes above 400 m^3^/ha can be found (Figure 2). Carbon stock changes show a scattered picture (figure 3). Especially regions with decreasing carbon stocks can be found in southern Germany, where initial carbon stocks were high. Similarly the development of the average stand ages is non-uniform (figure 4). Broadleaved species are dominating; only regions in southern and central Germany can be found with a high abundance of coniferous species (figure 5).

## Discussion

In climate change debates "adaptation" and "mitigation" are fundamental terms. While mitigation is related to the causes of climate change, adaptation is related to the effects of the phenomenon. Climate adaptation refers to the ability of a system to adjust to climate change. Climate mitigation is any action taken to permanently reduce or eliminate the long-term risk and hazards of climate change.

Despite the fact that forests were able to adapt to changing climatic conditions in past millennia, there will be regions where the current rate of changes in temperature and precipitation patterns will likely be beyond the inherent adaptive capacity of forests due to the long lifetime/longevity of trees and their long-term reproduction cycles [3,7,8]. This holds especially true for forest tree species that grow at the limits of their natural range [10]. Forest management offers the possibility to strengthen the adaptive capacity of forests by planned mitigation activities [32].

Our study focused on forest growth and related carbon stock changes. However, sustainable forest management has to address the multiple functions of forests demanded by various stakeholders, such as timber production, carbon sequestration, biodiversity, protection, or recreation [33]. A major concern is the potential loss of forest biodiversity due to climatic changes and forest management. Future forest management will be challenged to adjust forests to changing climatic conditions, to prevail sustainable and economic viable timber production, and to maintain and enhance forest biodiversity. The decision about the "optimal" forest management strategy has to address multiple criteria and seek for a reconciliation of interests in order to meet the different and partially contradictory stakeholder demands. For the example of Germany our study indicates that management schemes rather than climate change scenarios drive the future development of managed forests.

## Conclusions

What holds for our case study in Germany can be transferred to other regions, as forest management offers a wide range of instruments suitable to reduce the long-term risk and hazards of climate change to managed forests. Commercial forestry adaptations could include salvaging dead and dying timber and replanting species appropriate to a new climate. Available management strategies include options such as the selection of tree species and provenances adapted to future climate patterns, reduce the rotation cycle to speed the establishment of better adapted species, use of germplasm mixtures with high levels of genetic variation, or design and establish long-term multi species/seedlot trials to test improved genotypes across a diverse array of climatic environments [34].

## Methods

Future scenarios of forest development depend on the initial state of forests at a given point in time and the future development of external factors, i.e. management regimes and environmental factors. The focus of our study was the description of potential scenarios and not a prediction of future realities. This accommodates the fact that future climate conditions, land-use, economy or ecological capacity of trees to adapt to changing environmental conditions are unknown and can only be anticipated under specific constraints.

### Input data

We utilized the data from the German national forest inventory (NFI) to define the initial state of forests for the year 2000. The NFI is a sample based survey that provides a representative description of German forests [35]. The assessments are carried out on in-situ sample plots, but do not provide information on individual forest stands. For the simulation of forest management strategies we formed virtual forest stands based on plot level information for the distribution of tree species, tree dimensions such as tree height, stem diameters or timber volumes, tree age, and number of trees per hectare. In addition factors describing site quality (nutrient supply, soil moisture), topography, and growing regions were assigned to the virtual stands utilizing information from the German soil map [36], the digital terrain model from the Shuttle Radar Topography Mission (SRTM Level I) [37], and the German forest site classification [38].

### Management alternatives

Three management alternatives were selected to display the wide range of potential forest developments under different management objectives [27-30].

Where the management objective is maximum profit, the final cut is realized when a minimum age is reached (50 years for coniferous trees, 70 years for broadleaved trees) and the required rate of return becomes smaller than 2 percent. Equation 1 gives the decision rule for thinning; the required rate of return is calculated for a period of 5 years. As long as the required rate of return is above 2 percent for a five year period, the net wood revenue, A, decides whether a thinning takes place or not.

Equation 1:

$$5\sqrt{\frac{A_{t}+5}{A_{t}}}-1<0.0p$$

where:

A_t _net revenue at age t [€].

p interest rate

**Figure 6:**
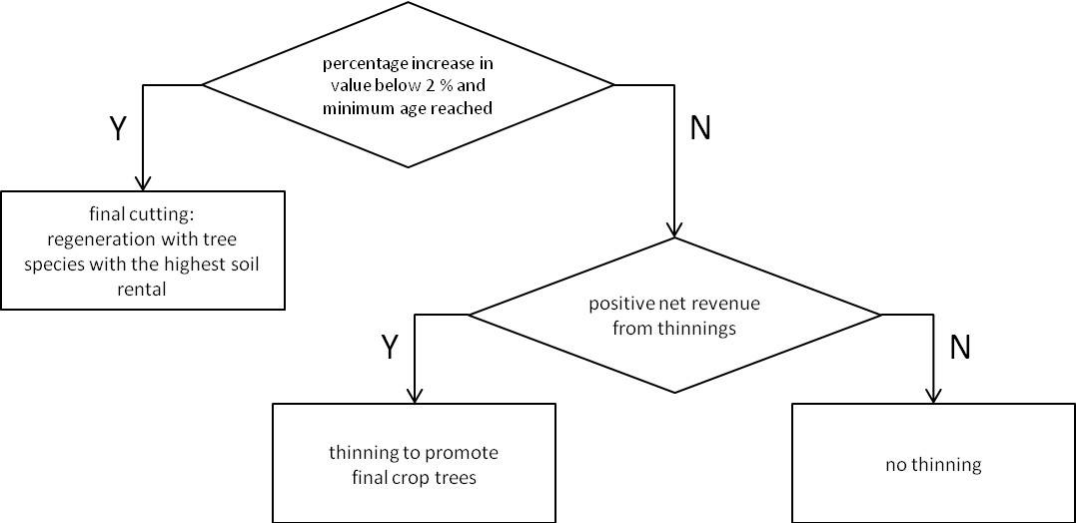
**Decision tree of management type "maximum profit"**.

After a final harvest the soil rental, sr, is calculated for different tree species (equation 2). The tree species yielding the highest soil rental, sr, is used for replanting (figure 6).

Equation 2:

$$sr_{\left(u,sp\right)}=\frac{0.0p*(A_{u}+\sum_{t=1}^{u}\left(D_{t}*1.0p^{u-t})-c*1.0p^{u}\right)}{1.0p^{u}-1}$$

where:

sr soil rental [€]

p interest rate

A_u _net revenue from final cutting at rotation period u [€]

D_t _net revenue from thinning at age t [€]

c planting costs at age t = 0 [€]

u rotation period

sp species

**Figure 7:**
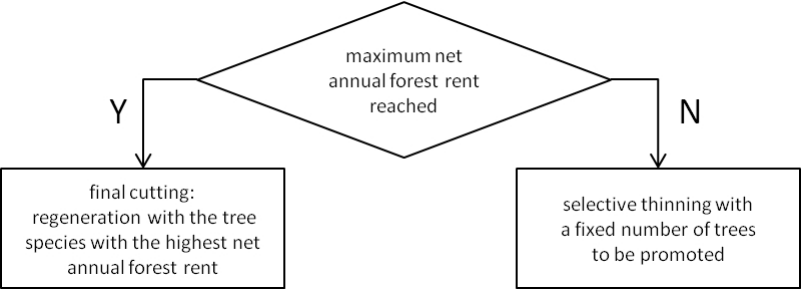
**Decision tree of management type "maximum net annual forest rent"**.

Under the management objective *maximum net annual forest rent *the decision about final cuts is based on the increase of the forest rent. As long as the forest rent is increasing forest stands are thinned but not finally cut. Until this point is reached selective thinning is performed. As soon as a forest stand reaches the phase of its highest value increment the final cutting is carried out. Reforestations after final cuts are realized with species that guarantee maximum net annual yield from the forest under the specific, local site conditions (figure 7).

In order to be able to determine whether the phase of maximum value increment is reached, the future value increment of a stand is simulated for a 250 year period with constant climatic conditions on the given site. The forest rent is calculated in five-year steps (equation 3) and the age of maximum value increment can be identified. The same approach is used for the determination of the tree species; the tree species with the highest expected net annual yield is used for regeneration.

Equation 3:

$$nfr_{\left(u\right)}=\frac{A_{u}+\sum_{t=1}^{u}D_{t}-c}{u}$$

where:

nfr_(u) _net annual forest rent at rotation period u [€]

A_u _net revenue from final cutting at rotation period u [€]

D_t _net revenue from thinning at age t [€]

c planting costs at age t = 0 [€]

**Figure 8:**
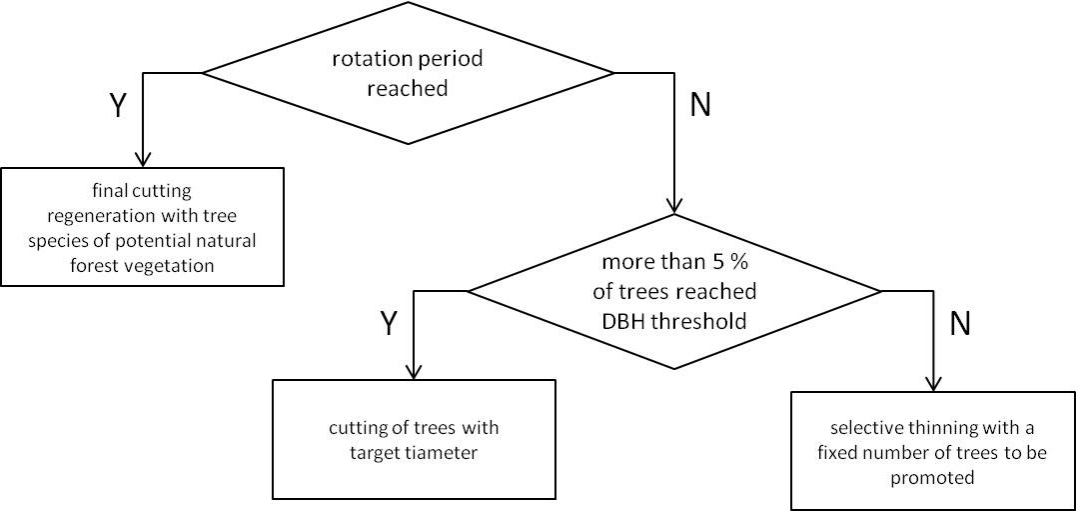
**Decision tree of management type "diameter limit cut"**.

Under the management objective *diameter limit cut *the decision for timber cuts is oriented towards biological criteria and reflects close-to-nature forest management. Trees are cut when they reach a threshold diameter at breast height (DBH), which is fixed between 45 cm for spruce and 80 cm for oak. When a given rotation period is reached, the whole stand is cleared and reforested with the species of the potential natural forest vegetation. In the meantime selective thinning is realized every five years (figure 8).

### Models

The statistical model WETTREG (German: Wetterlagenbasiertes Regionalisierungsverfahren. English: Weather pattern-based regionalization method) was applied to derive future climate change scenarios [39,40]. WETTREG was primarily developed for climate change impact studies and provides time series with high spatial resolution of climate parameters. The underlying algorithms use the statistical relationship between observations in local weather stations and large scale, atmospheric circulation patterns and expand those on simulation runs of the global climate model ECHAM5 [41].

**Figure 9:**
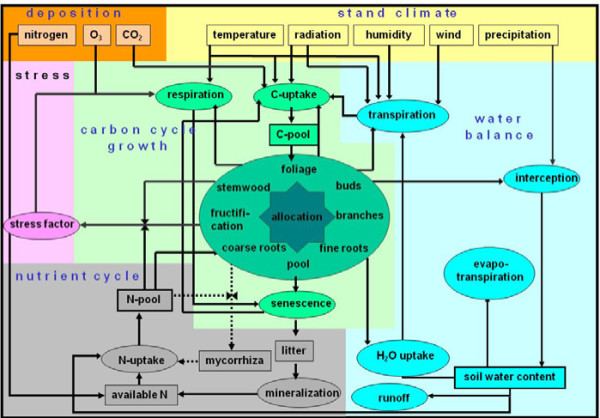
**Cycles and processes in Balance**.

The simulation of forest growth under changing climatic conditions and management regimes render the application of a growth model necessary, which factors in different tree species, forest stand structures, management regimes and a climate-sensitive reaction of tree growth and mortality. We chose the growth model SILVA [42], which is single-tree based and has been parameterized by utilizing single tree growth measurements of long-term experimental plots in Middle Europe. SILVA was originally developed for modelling volume growth but has recently been extended by a biomass module which provides biomass estimates for the individual tree compartments stem, root, bark, branches, and leaves by allometric functions [43]. The conversion of tree biomass estimates into estimates of carbon stock is straightforward. The purely statistical parameterization of SILVA was complemented by the eco-physiological growth model BALANCE [44,45]. BALANCE reproduces growth and allocation patterns via the availability of light, nutrients and water (figure 9). While SILVA models growth from single trees to forest enterprises for 5 year intervals, BALANCE provides growth for biomass compartments in 10-days or monthly resolution.

## List of abbreviations

BALANCE: physiological biomass model for trees; ECHAM5: Atmospheric General Circulation Model Version 5; GDP: Gross Domestic Product; ICP-Forests: International Co-operative Programme on Assessment and Monitoring of Air Pollution Effects on Forests; IPCC: International Panel on Climate Change; NFI: National Forest Inventory; SILVA: Forest Growth Simulator; SRES: Special Report on Emission Scenarios; SRTM: Shuttle Radar Topography Mission; WETTREG: Wetterlagenbasiertes Regionalisierungsverfahren.

## Competing interests

The authors declare that they have no competing interests.

## Authors' contributions

MK coordinated the research project underlying this paper and drafted the manuscript. RH, RK, KO and BK were responsible for the data procurement and developed the simulation suite. MD and MaK provided the economic rules for the management objectives. MA and FM contributed the modelling of soil carbon development. TM, HP and TR developed the growth models BALANCE and SILVA. All authors read and approved the final manuscript.
